# Acupuncture With *deqi* Modulates the Hemodynamic Response and Functional Connectivity of the Prefrontal-Motor Cortical Network

**DOI:** 10.3389/fnins.2021.693623

**Published:** 2021-08-16

**Authors:** Xiaopeng Si, Shaoxin Xiang, Ludan Zhang, Sicheng Li, Kuo Zhang, Dong Ming

**Affiliations:** ^1^Academy of Medical Engineering and Translational Medicine, Tianjin University, Tianjin, China; ^2^Tianjin Key Laboratory of Brain Science and Neural Engineering, Tianjin University, Tianjin, China; ^3^Tianjin International Engineering Institute, Tianjin University, Tianjin, China; ^4^Institute of Applied Psychology, Tianjin University, Tianjin, China

**Keywords:** acupuncture, functional connectivity, functional near-infrared spectroscopy, motor cortex, prefrontal cortex

## Abstract

As a world intangible cultural heritage, acupuncture is considered an essential modality of complementary and alternative therapy to Western medicine. Despite acupuncture’s long history and public acceptance, how the cortical network is modulated by acupuncture remains largely unclear. Moreover, as the basic acupuncture unit for regulating the central nervous system, how the cortical network is modulated during acupuncture at the Hegu acupoint is mostly unclear. Here, multi-channel functional near-infrared spectroscopy (fNIRS) data were recorded from twenty healthy subjects for acupuncture manipulation, pre- and post-manipulation tactile controls, and pre- and post-acupuncture rest controls. Results showed that: (1) acupuncture manipulation caused significantly increased acupuncture behavioral *deqi* performance compared with tactile controls. (2) The bilateral prefrontal cortex (PFC) and motor cortex were significantly inhibited during acupuncture manipulation than controls, which was evidenced by the decreased power of oxygenated hemoglobin (HbO) concentration. (3) The bilateral PFC’s hemodynamic responses showed a positive correlation trend with acupuncture behavioral performance. (4) The network connections with bilateral PFC as nodes showed significantly increased functional connectivity during acupuncture manipulation compared with controls. (5) Meanwhile, the network’s efficiency was improved by acupuncture manipulation, evidenced by the increased global efficiency and decreased shortest path length. Taken together, these results reveal that a cooperative PFC-Motor functional network could be modulated by acupuncture manipulation at the Hegu acupoint. This study provides neuroimaging evidence that explains acupuncture’s neuromodulation effects on the cortical network.

## Introduction

External neurostimulation and its modulatory effect on the cortical network have been the subject of extensive research by neuroscientists. As an external neuromodulation method that originated in ancient China, acupuncture balances sympathetic and parasympathetic nervous activity (Takahashi, 2011) and modulates the human brain (Yu et al., 2018). As a world intangible cultural heritage, acupuncture is now considered an essential mode of complementary and alternative therapy to Western medicine (Yoo et al., 2004). Studies have demonstrated that acupuncture has therapeutic effects on various neuropsychiatric disorders such as neuropathic pains (Miranda et al., 2015), Alzheimer’s disease (Liang et al., 2014), and Parkinson’s disease (Jiang et al., 2018). Besides acupuncture’s clinical therapeutic effects, it has been shown that acupuncture could enhance human cognition and memory (Zheng et al., 2018) and regulate emotion processing for healthy people (Hui et al., 2005). Despite acupuncture’s long history and public acceptance, the underlying neural mechanism of acupuncture’s neuromodulation effects on the cerebral cortex remain unclear.

The needling sensation that subjects experience during acupuncture is called *deqi* (Hui et al., 2007), which refers to the excitation of vital energy, known as *qi* in languages from China (Kong et al., 2007). Importantly, *deqi* is an indispensable requirement for achieving acupuncture efficacy according to Traditional Chinese Medicine (TCM) (Sun et al., 2020). Previous studies showed that acupuncture with *deqi* sensation could produce better therapeutic efficacy and reduce the severity of symptoms compared to no *deqi* sensation (Hui et al., 2007; Fang et al., 2009; Lee et al., 2017). Therefore, *deqi* was employed as a valuable behavioral index to evaluate the acupuncture effect (Yang et al., 2013). However, the current behavioral evaluation of *deqi* is subjective and insufficient. There is a lack of an objective neural biomarker for quantifying acupuncture’s *deqi* sensation.

The human brain is an integrative and complex network system (Bassett and Sporns, 2017; Cai et al., 2018), in which regions are considered as nodes, and functional or structural connectivity between regions is considered as edges (van den Heuvel and Pol, 2010). According to the network control theory in network neuroscience, the brain’s functional network could be modulated by various neuromodulation methods, such as acupuncture (Takahashi, 2011), transcranial magnetic stimulation (TMS) (Curtin et al., 2019b), transcranial electrical stimulation (tES) (Wang et al., 2019), and transcranial focused ultrasound (tFUS) (Legon et al., 2014).

During the acupuncture stimulation process, the afferent neural activities from the peripheral nervous system are firstly carried up to the brainstem and then are transferred to the subcortical regions in the central nervous system, such as the thalamus, cerebellum, amygdala (Zhang et al., 2012). Finally, the high-level cerebral cortex of the human brain, such as the prefrontal and sensorimotor network, is modulated by the afferent signals from subcortical regions (Lund and Lundeberg, 2016). By modulating the cortical network during acupuncture stimulation, the human brain’s cognition functions and behavioral outcomes are finally regulated (Zheng et al., 2018; Ghafoor et al., 2019). However, how the brain’s functional cortical network changes and what specific cortical regions are involved during acupuncture with *deqi* remain mostly unclear.

To reveal how the human brain’s functional network changes during acupuncture with *deqi*, different neuroimaging methods, such as functional magnetic resonance imaging (fMRI) and electroencephalography (EEG) have been used in previous studies. The high-spatial-resolution fMRI studies mainly revealed the involvement of several subcortical brain regions during acupuncture (Hui et al., 2005; Dhond et al., 2008; Fang et al., 2009; Asghar et al., 2010; Hui et al., 2010; Zhong et al., 2012). For example, it was found that the limbic-paralimbic-neocortical network, including the subgenual anterior cingulate cortex, posterior cingulate cortex, amygdala, and hippocampus, was deactivated during acupuncture (Dhond et al., 2008; Fang et al., 2009; Zhong et al., 2012), whereas the insula, thalamus, the somatosensory, and anterior cingulate cortex were activated by acupuncture (Dhond et al., 2008; Zhong et al., 2012). Besides, fMRI studies also revealed that the sub-cortical regions’ functional connectivity could be modulated during acupuncture (Dhond et al., 2008; Zhong et al., 2012). Previous fMRI studies have found that changes in the sub-cortical regions’ functional connectivity during acupuncture, but only a few studies focused on the cortical network changes, but how the cortical network is modulated during acupuncture with *deqi* remains unclear.

Meanwhile, scalp EEG was also used to explore the modulation effect of acupuncture, which found that the delta and theta frequency band’s EEG power (Yu et al., 2017, 2018) and functional connectivity were increased by acupuncture stimulation (Chen et al., 2006; Yu et al., 2017, 2019). However, due to the volume conduction effect (Hipp et al., 2012) and the low-spatial resolution (Tabar and Halici, 2017), EEG failed to reveal the specific cortical region’s involvement during acupuncture. In summary, how the key node and functional connectivity of the cortical network are modulated by acupuncture with *deqi* remains mostly unclear.

The functional near-infrared spectroscopy (fNIRS), a relatively novel non-invasive neuroimaging technique (Quaresima and Ferrari, 2019), is good at detecting the cerebral cortex’s hemodynamic responses, such as oxygenated hemoglobin (HbO) and deoxygenated hemoglobin (HbR) changes, which could reflect the underlying cortical neural activities according to the neurovascular coupling effect (Keles et al., 2016; Soltanlou et al., 2018). Compared with EEG, fNIRS has a much higher spatial-resolution and is especially good for detecting cortical neural activities (Wang et al., 2020). Moreover, compared to fMRI, fNIRS is both portable and less sensitive to motion artifacts. As a useful neuroimaging tool, fNIRS is widely used to investigate the high-level cerebral cortex’s hemodynamic activities and the related functional network in various neuromodulation studies, such as TMS (Park et al., 2017; Curtin et al., 2019b), tES (Patel et al., 2020), and acupuncture (Takamoto et al., 2010; Ghafoor et al., 2019; Rojas et al., 2019). Previous acupuncture studies also showed the prospect of applying fNIRS to study acupuncture’s neural mechanism (Takamoto et al., 2010; Ghafoor et al., 2019; Rojas et al., 2019). However, to our knowledge, few studies used fNIRS to simultaneously investigate the cortical response and cortico-cortical functional connectivity during acupuncture with *deqi*.

The Hegu acupoint (LI-4) is considered the basic neural acupuncture unit (Zhang et al., 2012) for regulating the central nervous system according to the TCM. For example, acupuncturing at the Hegu acupoint with *deqi* could relieve pain (Liang et al., 2014) and improve the human brain’s cognition (Zheng et al., 2018) by balancing the brain activity as a whole. In addition, previous studies have shown that acupuncture at the Hegu acupoint could regulate the activity of the brain, activating or inhibiting areas of the brain to achieve the effect of anesthetic (Hui et al., 2010; Liang et al., 2014). However, how the cortical network is modulated during acupuncture at Hegu acupoint is largely unclear. Considering that the human brain is an integrative and complex network system (Bassett and Sporns, 2017; Cai et al., 2018), we hypothesized that acupuncture at the Hegu acupoint with *deqi* could achieve its acupuncture effects by modulating the large-scale cortical network. The functional cortical network revealed by fNIRS could thus be used as an objective biomarker to quantify the behavior *deqi* effect for acupuncture.

To test our hypothesis, the multi-channel fNIRS data were recorded from twenty healthy subjects for acupuncture manipulation, pre- and post-manipulation tactile controls, and pre- and post-acupuncture rest controls. Meanwhile, each subject’s acupuncture *deqi* behavior performance was collected. As the basic acupuncture unit for regulating the central nervous system according to TCM (Zhang et al., 2012), the Hegu acupoint was employed in this study to explore the neuromodulation effects of acupuncture. To reveal the acupuncture with *deqi* responsive areas, the hemodynamic responses and the power of HbO concentration were computed and compared between different conditions. To investigate the correlation relationship between hemodynamic response and behavior performance for acupuncture with *deqi* responsive areas, Pearson’s correlation was analyzed. To reveal how the cortical network is modulated by acupuncture with *deqi*, the functional connectivity and graph theory parameters of the network were calculated and compared between acupuncture manipulation and controls.

## Materials and Methods

### Participants

Twenty healthy right-handed volunteers (23.9 ± 1.5 years old (mean ± std), age range from 22 to 28 years, twelve males and eight females) were recruited for the acupuncture experiment (Table 1). All subjects have normal intelligence, no history of mental illness, and no brain damage caused by long-term medication. Most importantly, all subjects in this study were naive to acupuncture, having never experienced an acupuncture treatment. After confirming that each subject met all the inclusion criteria, subjects gave written informed consent prior to participating, which was reviewed by the Institutional Review Board and Ethics Committee of Tianjin University.

**TABLE 1:** Detailed information of subjects.

Subject	Age (years)	Gender	Accepted channels/All	Accepted (>50% channels accepted)
S1	28	Male	35/48	Yes
S2	23	Male	35/48	Yes
S3	23	Female	30/48	Yes
S4	25	Male	46/48	Yes
S5	24	Male	33/48	Yes
S6	23	Female	35/48	Yes
S7	22	Male	47/48	Yes
S8	23	Male	34/48	Yes
S9	27	Male	46/48	Yes
S10	23	Female	34/48	Yes
S11	24	Male	46/48	Yes
S12	22	Male	38/48	Yes
S13	23	Male	25/48	Yes
S14	25	Male	22/48	No
S15	24	Female	47/48	Yes
S16	23	Female	34/48	Yes
S17	24	Male	34/48	Yes
S18	23	Female	15/48	No
S19	23	Female	46/48	Yes
S20	26	Female	13/48	No

### Acupuncture Experiment Paradigm

**FIGURE 1:**
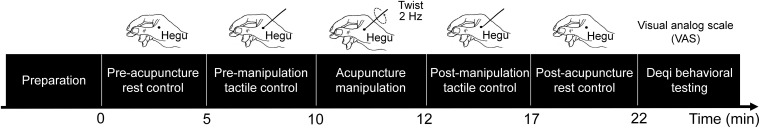
Acupuncture experiment paradigm. During the preparation, subjects were asked to be familiar with the *deqi* behavioral questionnaire evaluated by the visual analog scale (VAS). There are five conditions in the experiment: pre-acupuncture rest control (5 min), pre-manipulation tactile control (5 min), acupuncture manipulation (2 min), post-manipulation tactile control (5 min), and post-acupuncture rest control (5 min). After the experiment, subjects were asked to evaluate the needling sensations for the pre-manipulation tactile control, acupuncture manipulation, and post-manipulation tactile control. The twisting frequency in acupuncture manipulation is 2 Hz.

**FIGURE 2:**
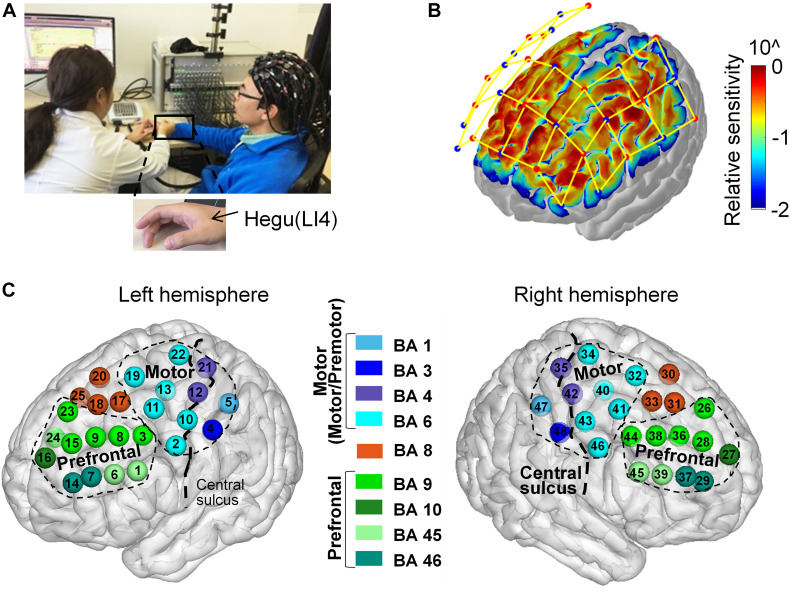
Acupuncture experiment, fNIRS channel configuration, and anatomical location. **(A)** Acupuncture experiment. An experienced acupuncturist conducted acupuncture on the subject wearing a 48 channel fNIRS measuring cap. The black arrow indicated the location of the Hegu acupoint (LI-4). **(B)** fNIRS channel configuration. Red dots represented light sources, and blue dots represented detectors. Each fNIRS channel’s relative cortical sensitivity was visualized by pseudo-color. **(C)** Anatomical location. Each channel’s Brodmann label was indicated by a different color. All channels were divided into three regions of interests (ROIs), the prefrontal cortex (PFC, colored with the green series system), the motor-related area (motor and premotor cortex, colored with the blue series system), and the remaining Brodmann area 8 (BA 8, colored with red, BA, Brodmann area).

The acupuncture manipulation was performed by an experienced acupuncturist. The acupuncture site was the Hegu acupoint, also known as “Large intestine 4” (LI4) at the midpoint on the radial side of the second metacarpal (Kong et al., 2015; Figure 1). Throughout the experiment, all subjects were asked to sit in a comfortable chair during the entire process of the acupuncture experiment in a quiet room (Figure 2A). All subjects were required to keep relaxed and to keep their heads still during the experiment.

During the preparation, each subject was asked to familiarize themself with the *deqi* sensation behavior questionnaire, containing six individual visual analog scales (VAS) for evaluating the multiple acupuncture *deqi* sensations (Kou et al., 2007), which including *suan* (aching or soreness), *ma* (numbness or tingling), *zhang* (fullness/distention or pressure), *tong* (dull pain), *zouchuan* (spreading, a feeling of extension from the acupuncture position to the arm) and *zhong* (heaviness) according to TCM (Asghar et al., 2010; Hui et al., 2011; Yu et al., 2012). The intensity of each needing sensation was rated by a point between 0 (none) and 10 (strongest) to quantify each of the six sensations for each subject.

Considering that the quasi-experimental design could involve the real-world acupuncture intervention instead of artificial experimental settings with high external validity (Bärnighausen et al., 2017), the quasi-experimental design was employed in this study (Figure 1), which was also used in previous acupuncture studies (Yu et al., 2017; Yu et al., 2018). The whole acupuncture procedure included the following five conditions (Figure 1). (I) Pre-acupuncture rest control: each subject was asked to keep awake and rest for 5 min without movement before the needle was inserted by the acupuncturist. (II) Pre-manipulation tactile control: a single-use sterile acupuncture needle was inserted by the acupuncturist at the Hegu acupoint to a certain depth without twisting. To avoid the bias of acupuncture site, random acupuncture on the right or left hand at the Hegu acupoint was conducted in this experiment. During this condition, each subject was asked to maintain awake and rest for 5 min before the acupuncture twisting. (III) Acupuncture manipulation: the subjects were acupunctured by using the twirling method at the Hegu acupoint for 2 min. The twisting is mainly manipulated by the thumb to push forward with force, which was within a range of 90°–180° (Yu et al., 2017) with a frequency of 2 Hz. (IV) Post-manipulation tactile control: after the acupuncture manipulation period, each subject was asked to keep awake in a resting state with a needle inserted for 5 min. During this condition, the needle was kept at the Hegu acupoint without the twist. (V) Post-acupuncture rest control: after the acupuncturist removed the needle, each subject was asked to maintain awake and rest for 5 min without movement. Immediately after the acupuncture experiment, the behavior questionnaire was given to each subject to evaluate his/her *deqi* sensation for the following three conditions: pre-manipulation tactile control, acupuncture manipulation, post-manipulation tactile control.

### fNIRS Data Acquisition

The fNIRS measurements were conducted by a multi-channel fNIRS system (NirScan, Danyang Huichuang Medical Equipment Co., Ltd.) at a sampling rate of 9 Hz. Near-infrared light of three different wavelengths (740, 808, and 850 nm) was used to detect the concentration signals of HbO and HbR. For the channel configuration, 15 light sources and 16 detectors were plugged into a holder and resulted in 48 measurement channels, which were positioned by referring to the standard international 10–20 system of electrode placement (Figure 2B). The distance between the light source and the detectors was 3 cm.

### fNIRS Channel Location and Cortical Sensitivity

To obtain the Montreal Neurological Institute (MNI) coordinates of each fNIRS channel, the spatial coordinates of the light sources, detectors, and anchor points (located at Nz, Cz, Al, Ar, Iz referring to the standard international 10–20 system of electrode placement) were firstly measured by using the electromagnetic 3D digitizer system (FASTRAK. Polhemus VT, United States). Then, the spatial coordinates of the light sources and detectors were registered to the standard MNI space by using the spatial registration approach (Tsuzuki et al., 2007). Thirdly, the MNI coordinate of the midpoint between a light source and detector pair was determined, as each fNIRS channel consisted of a light source and detector pair. Fourthly, the midpoint was spatially projected to the cortex surface of the MNI “Colin27” brain template, and the fNIRS channel’s coordinate was defined as the MNI coordinate of the cortical projected point. Finally, to reduce the measurement error, the coordinates of left/right hemispheric symmetric channels were averaged and marked with positive or negative hemispheric information (Table 2). To reveal the probability of the transmitted photon path in the cortex, Monte-Carlo photon transmitted software tMCimg was used to obtain the cortical sensitivity (Boas et al., 2002). Then the cortical sensitivity of each channel was displayed on the MNI “Colin27” brain template by using the AtlasViewer (Aasted et al., 2015; Figure 2B). The cortical sensitivity information of each channel was then used to visualize different indexes, such as statistical significance, hemoglobin concentration, and channel label.

**TABLE 2:** The MNI coordination and Brodmann label of each channel.

Channel	X	Y	Z	Brodmann label	ROI
Ch1/Ch45	−54/54	31	27	BA 45	Prefrontal
Ch2/Ch46	−59/59	8	39	BA 6	Motor
Ch3/Ch44	−46/46	22	44	BA 9	Prefrontal
Ch4/Ch48	−60/60	−16	44	BA 3	Motor
Ch5/Ch47	−54/54	−31	55	BA 1	Motor
Ch6/Ch39	−47/47	42	25	BA 45	Prefrontal
Ch7/Ch37	−43/43	55	25	BA 46	Prefrontal
Ch8/Ch38	−41/41	35	45	BA 9	Prefrontal
Ch9/Ch36	−31/31	44	43	BA 9	Prefrontal
Ch10/Ch43	−54/54	−3	51	BA 6	Motor
Ch11/Ch41	−44/44	13	58	BA 6	Motor
Ch12/Ch42	−45/45	−16	62	BA 4	Motor
Ch13/Ch40	−32/32	−3	62	BA 6	Motor
Ch14/Ch29	−32/32	60	19	BA 46	Prefrontal
Ch15/Ch28	−23/23	55	39	BA 9	Prefrontal
Ch16/Ch27	−14/14	67	30	BA 10	Prefrontal
Ch17/Ch33	−29/29	26	60	BA 8	BA8
Ch18/Ch31	−21/21	36	58	BA 8	BA8
Ch19/Ch32	−24/24	13	71	BA 6	Motor
Ch20/Ch30	−5/5	24	67	BA 8	BA8
Ch21/Ch35	−34/34	−29	71	BA 4	Motor
Ch22/Ch34	−26/26	−16	79	BA 6	Motor
Ch23/Ch26	−10/10	49	53	BA 9	Prefrontal
Ch24	2	50	34	BA 9	Prefrontal
Ch25	3	32	54	BA 8	BA8

*BA, Brodmann area, ROI, region of interest.*

### Brodmann Atlas (BA) Label and Regions of Interest (ROI)

To obtain the BA label of each channel, the MNI-to-BA Tool (Okamoto et al., 2009) was used. Firstly, a 10 mm radius sphere with each fNIRS channel’s MNI coordinate as the center was determined. Secondly, all possible BA labels within the sphere were determined. Then, the percentage of each BA label’s voxel number within the sphere over the entire sphere’s voxel number was calculated. Thirdly, the BA label with the highest percentage was identified as the BA label of a given fNIRS channel. Finally, each fNIRS channel and the corresponding BA label were visualized on the “Colin27” brain template by using BrainNet Viewer (Xia et al., 2013; Figure 2C). According to each fNIRS channel’s BA label and the anatomical location information from the “Colin27” brain template, all fNIRS channels were divided into the following three ROIs, including the motor area, the prefrontal cortex (PFC) area, and the remaining Brodmann area 8 (Figure 2C).

### The Acupuncture’s Behavioral *deqi* Index Calculation and Statistical Analysis

To quantify the acupuncture’s *deqi* behavioral effect, the Massachusetts general hospital acupuncture sensation scale (MASS) was administered as the *deqi* index to quantify the needling sensation in this study (Kong et al., 2007; Yu et al., 2012). According to the MASS index, the VAS scores for all the different needling sensations were sorted from highest to lowest for each subject. The *deqi* index was calculated according to the following equation:


$$deqi\begin{array}{c} \mathrm{index} \end{array}=\frac{\sum_{i=1}^{n}{\left(1/2\right)}^{i}R_{i}}{1-{\left(1/2\right)}^{n}},$$

where *R*_*i*_ indicates the highest to lowest ranking index of *i th* VAS score, and *n* represents the number of different *deqi* sensations on the behavior questionnaire. The *deqi* index was calculated for each subject (Supplementary Table 1).

**FIGURE 3:**
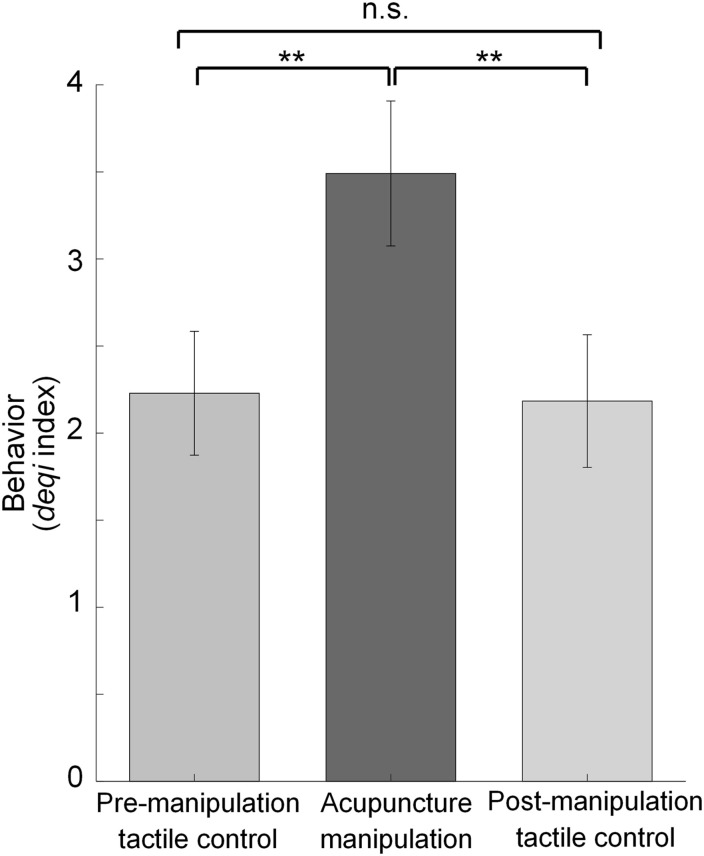
Statistical comparisons of behavior *deqi* index for different conditions. Behavior results comparison for pre-manipulation tactile control vs. acupuncture manipulation vs. post-manipulation tactile control. (mean ± SEM, paired *t*-tests with FDR correction for multiple comparisons, *n* = 17, **p < 0.01 and statistical power >0.95, n.s. for non-significance).

To evaluate if there is a significant *deqi* sensation difference between different conditions, paired *t*-tests were used on all subjects’ *deqi* data for tactile controls (averaging two tactile controls) vs. acupuncture manipulation (Figure 2A), and for pre-manipulation tactile control vs. post-manipulation tactile control (Figure 3), respectively. The *p*-values were corrected with false discovery rate (FDR) correction for multiple comparisons to minimize the risk of type I errors. To verify the validity of the statistical results, the statistical power was also calculated by using the free software G^∗^power.

### fNIRS Data Pre-processing

Because the fNIRS recorded raw data contained various noises, fNIRS raw data was pre-processed with the MATLAB^TM^ 17.0 (MathWorks, United States) software by the following procedure to improve the signal-to-noise ratio (SNR) (Supplementary Figure 1). Firstly, noisy channels were rejected if any of the following situations were met: (I) The optical intensity (OI) range exceeded the 0.5–1,000; (II) The source-detector separation range exceeded the range from 0 to 45; and (III) The ratio of the mean and standard deviation of the optical intensity [mean(OI)/std(OI)] is less than 2 (Huppert et al., 2009; Peters et al., 2020). Secondly, for a given subject, if the percentage of the subject’s rejected channels over all channels exceeds 50%, the subject with poor signal quality was also rejected, which was in line with the previous fNIRS study (Hakuno et al., 2020). In total, there were three subjects (S14, S18, S20) excluded from the subsequent analysis (Table 1). Thirdly, after excluding the noisy channels and subjects, the optical intensity raw data were converted into optical density (OD) (Supplementary Figure 1A).

To further improve the SNR of HbO and HbR, the following four pre-processing steps were applied (Supplementary Figure 1). In step 1, motion artifacts containing in the OD signals were corrected by using principal component analysis (PCA) (Supplementary Figure 1B; Behrendt et al., 2018; Di Lorenzo et al., 2019). In step 2, to remove various physiological noises such as cardiac (∼1 Hz), respiration (∼0.3 Hz), and low-frequency drifts (<0.09 Hz), the OD data were filtered by a 0.01–0.2 Hz Butterworth bandpass filter (Scholkmann et al., 2014; Chen and Glover, 2015; Pinti et al., 2019), which is in line with previous fNIRS studies (Zuo et al., 2010; Scholkmann et al., 2014; Chen and Glover, 2015). The Mayer wave (∼0.1 Hz) was removed by using a 0.1 Hz notch filter (Supplementary Figure 1C; Yucel et al., 2016). In step 3, the OD data were converted into HbO and HbR concentrations by applying the modified Beer-Lambert law (Supplementary Figure 1D; Scholkmann et al., 2014). In step 4, to further extract the functional brain activity, the hemodynamic modality separation (HMS) algorithm was used and the neural activity related HbO and HbR were obtained (Supplementary Figure 1E; Yamada et al., 2012).

### HbO Power Calculation of Each Epoch

After removing the motion artifacts and systemic noise by the pre-processing procedure, the HbO and HbR concentration with high SNR were obtained. Considering that HbO concentration proved to be more reliable and sensitive than HbR in the previous studies (Strangman et al., 2002), HbO signals were used for further analysis. Considering that the root-mean-square (RMS) amplitude of the time domain signal represented the integrated amplitude of major frequencies (Chuang et al., 2008), the RMS of HbO concentration was calculated as HbO power to quantify the cortical response intensity during acupuncture by the following steps. Firstly, the sliding window technique with the moving window length of 5 s without overlap was used to divide the HbO into epochs (Baczkowski et al., 2017). Secondly, to remove the outlier epoch and improve the SNR, the noisy outlier epoch was rejected if its absolute Z-score of HbO concentration was larger than 3. Finally, the HbO power of each epoch was calculated by using the RMS method (Supplementary Figure 1F).

### Hemodynamic Responses Analysis

**FIGURE 4:**
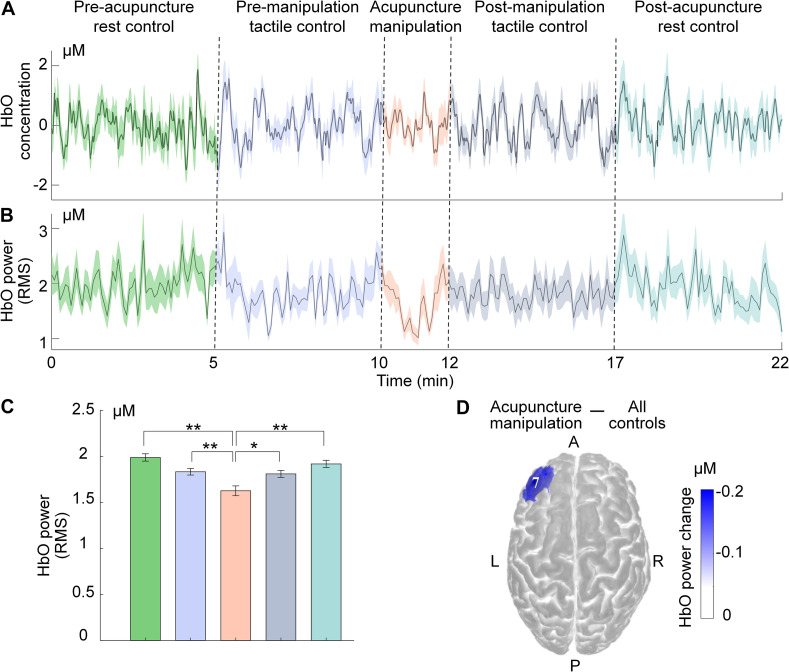
Decrease of HbO power during acupuncture manipulation on a typical channel of PFC. **(A,B)** Time series of group-level subjects’ averaged HbO concentration and HbO power under different conditions (mean ± SEM; subjects, *n* = 17, solid line for group-level averaged value, filled color for SEM). **(C)** Statistical comparisons of group-level subjects’ averaged HbO power for different conditions (mean ± SEM, one-way ANOVA, post-hoc *t*-tests with FDR correction for multiple comparisons, **p < 0.01 and statistical power >0.95, *p < 0.05 and statistical power >0.8). **(D)** Visualization of the typical channel’s HbO power change between acupuncture manipulation and all controls (averaging four control sets data).

To compare the differences between different conditions, averaged mean time series of HbO concentration and HbO power were computed across all subjects, which was also visualized by a typical channel (Figures 4A,B,D). To further assess if there existed significant difference for different conditions, the one-way analysis of variance (ANOVA) was conducted on the HbO power data with different conditions (pre-acupuncture rest control, pre-manipulation tactile control, acupuncture manipulation, post-manipulation tactile control, and post-acupuncture rest control) as a factor (Figure 4C), in which the data samples were each condition’s HbO power data of all epochs across all subjects. The post-hoc *t*-tests were conducted when the main effects or interactions in the ANOVA were significant. To minimize the risk of type I errors, the *p*-values were corrected with FDR correction for multiple comparisons. To verify the validity of the statistical results, the statistical power was also calculated by using the free software G^∗^power. The above statistical analysis was computed using MATLAB^TM^ 17.0 (MathWorks, United States) software.

### Statistical Mapping for Acupuncture Responsive Area

**FIGURE 5:**
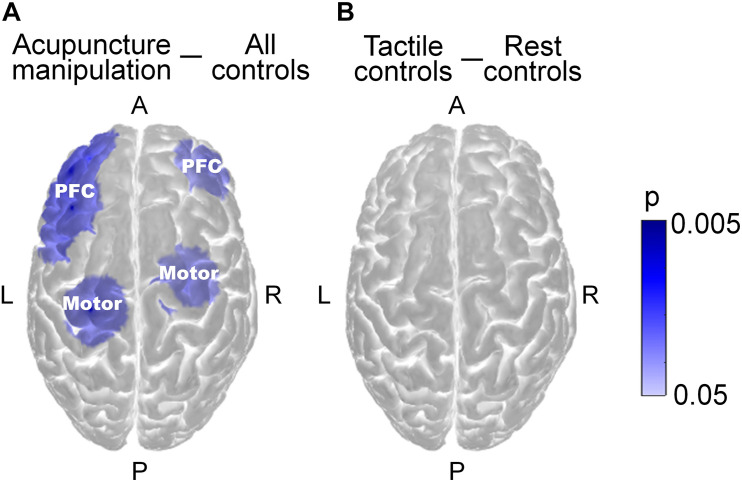
Across-condition statistical comparisons of HbO power on the group level. **(A)** Comparisons for acupuncture manipulation vs. all controls (combining four control groups). Channels with significant HbO power change were defined as acupuncture responsive channels. **(B)** Comparisons for tactile controls (combining two tactile controls) vs. rest controls (combining two rest controls) (Two-sample *t*-tests, FDR correction for multiple comparisons, alpha = 0.05 and statistical power >0.95, visualized by *p*-value, blue color for decrease).

To investigate the significant acupuncture responsive areas at the group level, the HbO power data of all epochs within different conditions across all subjects were taken as samples for statistical analysis. Considering that noisy outlier epochs were rejected previously and that the duration of different conditions varied, the data sample size of different conditions was unbalanced. Therefore, two-sample *t*-tests were conducted on the HbO power data between acupuncture manipulation and all controls for each channel. To minimize the risk of type I errors, the *p*-values were corrected with FDR correction for multiple comparisons and were visualized on the standard brain template (Figure 5A). The set of significant acupuncture responsive channels (*p* < 0.05) were defined as acupuncture responsive areas (Supplementary Figure 2A) that could be divided into the PFC area and motor area according to the label of each channel. As a comparison, two-sample *t*-tests were also conducted between tactile controls and rest controls (Figure 5B). In addition, to map the hemodynamic responses strength during acupuncture, the difference of the group averaged HbO power between acupuncture manipulation and all controls was calculated as the HbO power change. To verify the validity of the statistical results, the statistical power was also calculated by using the free software G^∗^power. Then, HbO power change was visualized for each channel on the standard brain template (Supplementary Figure 2B).

### Correlation Analysis Between Hemodynamic Responses and Behavioral Performances

To reveal the relationship between hemodynamic responses and acupuncture behavior performances for the acupuncture responsive areas, Pearson’s correlation and linear regression were performed between the *deqi* behavior data and HbO power change for all acupuncture significant responsive channels from the PFC and motor areas, respectively. To verify the validity of the correlation results, the statistical power was also calculated by using the free software G^∗^power. The previously rejected noisy channels were not included during the correlation analysis. For the linear regression, the 95% confidence intervals for the mean of the polynomial evaluation were computed and visualized (Supplementary Figure 3).

### Functional Connectivity and Statistical Analysis

To investigate the information flows in significant acupuncture responsive areas, functional connectivity analysis was conducted using the following steps.

Step 1, to obtain the subject level channel-to-channel functional connectivity, the significant acupuncture responsive channels were paired off to calculate the Pearson’s correlation coefficients for each subject (Zhang et al., 2011). The whole time series’ HbO concentration of acupuncture manipulation and all controls were used in the functional connectivity analysis, respectively. The previously rejected noisy channels were not included during this analysis.

Step 2, to further determine the significant channel-to-channel functional connectivity for each subject, a non-parametric permutation test was used as follows (van den Heuvel et al., 2019). Each channel’s HbO concentration time series data were divided into 2 s epochs for each subject’s acupuncture manipulation and all controls. Then, a 1,000-times permutation resampling method (the channel pairs’ corresponding epochs were shuffled randomly) to determine the significant functional connectivity (*p* < 0.001).

Step 3, each subject’s significant functional connectivity data were then subjected to Fisher’s r-to-z transformation to yield variants from the normal distribution (Ghafoor et al., 2019). To obtain the group level’s channel-to-channel functional connectivity matrices, then *z*-values were averaged across all subjects, and the group-level *z*-values were converted back into *r*-values via Fisher’s z-to-r inverse transformation, which was in line with previous studies (Zhu et al., 2017; Cai et al., 2018; Supplementary Figures 4A,B).

**FIGURE 6:**
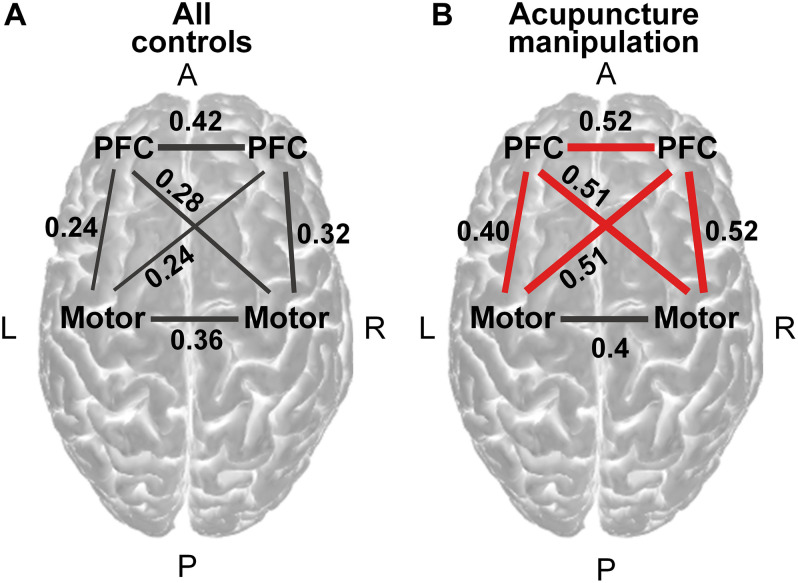
Functional connectivity analysis across all acupuncture with *deqi* responsive channels on the group level. **(A)** Region-to-region functional connectivity under all controls (averaging four controls, permutation test, *p* < 0.001). **(B)** Region-to-region functional connectivity under acupuncture manipulation (permutation test, *p* < 0.001). Red line indicates the significantly increased connections of acupuncture manipulation compared with all controls (paired *t*-tests, FDR corrected, alpha = 0.05 and statistical power >0.95). The magnitude of the connectivity value was indicated by the line width. PFC = prefrontal cortex.

Step 4, to obtain the group level region-to-region functional connectivity, subject level region-to-region functional connectivity data were firstly computed by averaging *r*-values of pair-wise channels within the same region by the previous Fisher’s r-to-z and z-to-r transformation method. Then, the group level region-to-region functional connectivity data were calculated by averaging the region-to-region functional connectivity data of all subjects, which were finally visualized on the standard brain template (Figures 6A,B and Supplementary Figures 4C,D).

Step 5, to reveal the significant group level region-to-region functional connectivity between acupuncture manipulation and all controls, paired *t*-tests were conducted on subject level channel-to-channel functional connectivity data within the same region across all subjects. To verify the validity of the statistical results, the statistical power was also calculated by using the free software G^∗^power. Significant increased region-to-region functional connectivity was visualized as a red line (Figure 6B).

Step 6, to further compare the functional connectivity changes of different region pairs between acupuncture manipulation and all controls (Supplementary Figure 5), paired *t*-tests were conducted on subject level channel-to-channel functional connectivity data within different region pairs (PFC-Motor, PFC-PFC, and Motor-Motor) across all subjects.

### Network Topological Property Analysis

After calculating each subject’s correlation coefficients for the pair-wise channels from the acupuncture responsive areas, the correlation coefficients matrix was obtained for each subject. Based on the calculated correlation coefficients matrix, the functional brain network could be reconstructed between acupuncture manipulation and all controls for each subject, in which channels served as nodes and functional connectivity between channels served as edges. The previously rejected noisy channels were not included during this analysis.

**FIGURE 7:**
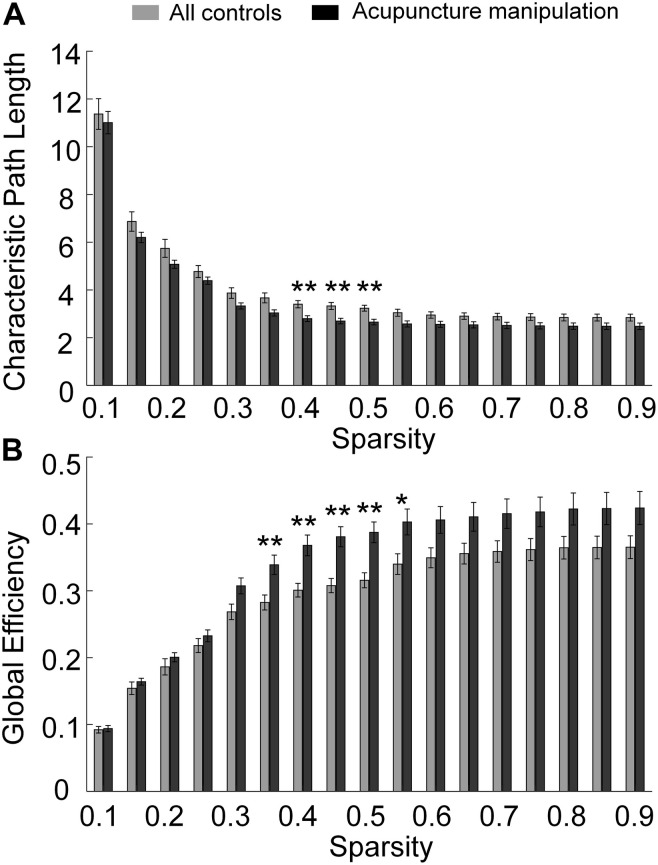
Statistical comparations of global network metrics for acupuncture manipulation vs. all controls with different sparsity thresholds. **(A)** Shortest path length. **(B)** Global efficiency. (paired *t*-tests, **p < 0.01 and statistical power >0.95, *p < 0.05 and statistical power >0.8, subjects, *n* = 17, sparsity range = [0.1, 0.9], sparsity step-size = 0.05).

To further reveal the acupuncture’s modulation effect on the functional brain network, the graph theory was utilized to describe the functional network’s topological structure changes between different conditions (Yu et al., 2018, 2019). The shortest path length and global efficiency were calculated and used as network metrics for each subject, in which the correlation coefficients matrix was thresholded over a range of sparsity (from 0.1 to 0.9 with step-size of 0.05) to investigate the relationship between network metrics and sparsity (Figure 7; Niu et al., 2013; Geng et al., 2017). In addition, other network metrics, including the local efficiency, clustering coefficient, and small-world, were also calculated, presented in Supplementary Figure 6. These network metrics were computed by a freely available MATLAB^TM^ toolbox, GRaph thEoreTical Network Analysis (GRETNA) (Wang et al., 2015).

For the network with N nodes and K edges, the shortest path length of the network (Figure 7A) was defined as the average of the shortest path lengths between all pairs of nodes in the network (Latora and Marchiori, 2003):


$$L_{p}=\frac{1}{N\left(N-1\right)}\sum_{i\neq j\in G}d_{ij},$$

where *d*_*ij*_ was the shortest path length between node *i* and node *j*, which meant the minimum number of edges included in the path that connected these two nodes.

The global efficiency (Figure 7B) was defined as (Latora and Marchiori, 2001):


$$E_{glob}=\frac{1}{N\left(N-1\right)}\sum_{i\neq j\in G}\frac{1}{d_{ij}},$$

where *d*_*ij*_ is the shortest path length between node *i* and node *j*.

To reveal the significant difference between acupuncture manipulation and all controls, paired *t*-tests were performed on all subjects’ global network metric data for different conditions (Figure 7). To verify the validity of the statistical results, the statistical power was also calculated by using the free software G^∗^power.

## Results

### Behavior *deqi* Performances Under Different Conditions

To verify the behavioral effectiveness of acupuncture manipulation, paired *t*-tests with FDR correction were used to compare the *deqi* index for different conditions (Figure 3 and Supplementary Table 1). The subjects’ *deqi* index of acupuncture manipulation was significantly larger than that of pre-manipulation tactile control and post-manipulation tactile control (^∗∗^*p* < 0.01 and statistical power >0.95) (Figure 3). In comparison, there was no significant difference between pre-manipulation tactile control and post-manipulation tactile control (n.s.) (Figure 3). These results indicated that acupuncture manipulation could cause significant *deqi* behavioral effects compared to tactile controls.

### Decreased HbO Responses by Acupuncture Manipulation

To reveal the *deqi* modulation effects of acupuncture on the cerebral cortex, the HbO concentration was firstly calculated after pre-processing of fNIRS data. Subject-averaged group-level results showed reduced amplitude of fluctuation for HbO concentration during acupuncture manipulation, which was shown by a typical channel from PFC (Figure 4A). Furthermore, the RMS of HbO concentration was computed as the HbO power index to quantify the HbO fluctuation energy. The typical channel’s subject-averaged results also showed reduced HbO power during acupuncture manipulation (Figure 4B). Furthermore, one-way ANOVA on the HbO power shown a significant difference between the five conditions (Figure 4C). The post-hoc *t*-tests with FDR correction shown that acupuncture manipulation’s HbO power was significantly smaller than the other four controls [pre-acupuncture rest control, ^∗∗^*p* < 0.01 and statistical power >0.95; pre-manipulation tactile control, ^∗∗^*p* < 0.01 and statistical power >0.95; post-manipulation tactile control, *p* < 0.05 and statistical power >0.8; post-acupuncture rest control, ^∗∗^*p* < 0.01 and statistical power >0.95] (Figure 4C). This typical channel’s HbO power change between acupuncture manipulation and all controls was computed and visualized on the standard brain template (Figure 4D).

### Acupuncture Responsive Area

To reveal the acupuncture with *deqi* responsive areas, the HbO power response patterns were examined across all channels from seventeen subjects (Figure 5A and Supplementary Figure 2). Channels from the bilateral PFC and motor cortex showed the strongest decreased HbO power change (Supplementary Figure 2B). Among them, the acupuncture responsive areas were identified as those channels that had significantly lower HbO power between acupuncture manipulation and all controls (acupuncture responsive channels, *n* = 8; *p* < 0.05; two-sample *t*-tests with FDR correction). The acupuncture with *deqi* responsive channels were distributed over the PFC and the motor cortex bilaterally, which was shown on the standard brain template (Figure 5A) and was indicated with channel labels (Supplementary Figure 2A). For the two different controls, there was no significantly changed HbO power channel by comparing the HbO power change between tactile controls and rest controls (Figure 5B).

### The PFC’s Hemodynamic Response Had a Positive Correlation Trend With Behavior Score

To investigate whether there is a relationship between hemodynamic response and behavior performance for acupuncture with *deqi* responsive areas, Pearson’s correlation was performed across all subjects for the bilateral PFC and the bilateral motor cortex, respectively. Interestingly, the HbO power change (acupuncture manipulation – all controls) of the acupuncture with *deqi* responsive channels from the PFC had a positive correlation trend with acupuncture behavior *deqi* index (*r* = 0.234, *p* < 0.05 and statistical power >0.50) (Supplementary Figure 3A). In contrast, there was no significant correlation for the acupuncture responsive channels from the motor cortex (*r* = −0.073, n.s.) (Supplementary Figure 3B). These results showed that the PFC’s HbO responses could predict the subject’s behavior performance during acupuncture manipulation.

### The PFC Related Connection Showed Significantly Increased Functional Connectivity

To reveal the information flows among key nodes in the network, functional connectivity analysis was used to explore the neural interactions between the acupuncture with *deqi* responsive channel pairs. The significant functional connectivity edges (permutation test, *p* < 0.001) were calculated and visualized for all controls (Figure 6A and Supplementary Figures 4A,C) and acupuncture manipulation (Figure 6B and Supplementary Figures 4B,D). It was shown that there was overall increased functional connectivity strength for acupuncture manipulation, compared with all controls (Figures 6A,B). Importantly, channels over the PFC were found to have significantly enhanced connections with the motor and the PFC under acupuncture manipulation compared with all controls (paired *t*-tests, alpha = 0.05 and statistical power >0.95) (Figure 6B), which was also verified by subsequent results.

Compared with all controls, PFC-Motor and PFC-PFC showed significantly higher functional connectivity strength under acupuncture manipulation (paired *t*-tests, ^∗∗^*p* < 0.01 and statistical power >0.95) (Supplementary Figures 5A,B). In contrast, the Motor-Motor connection showed no significant difference between acupuncture manipulation and all controls (paired *t*-tests, n.s.) (Supplementary Figure 5C).

### Topological Properties of the Cortical Functional Network

To further reveal acupuncture with *deqi*’s modulation effects on the functional brain network, graph theory analysis was performed on the functional connectivity network with the acupuncture with *deqi* responsive channels as nodes. The topological properties of the network were revealed by different global network metrics with different sparsity thresholds (Figure 7). To reveal the information transmission ability of the network, the shortest path length and global efficiency were showed. Compared with all controls, the shortest path length parameter showed a lower tendency. It was significantly lower within 0.4–0.5 sparsity range for acupuncture manipulation compared with all controls (paired *t*-tests, alpha = 0.05 and statistical power >0.95) (Figure 7A). On the contrary, the global efficiency parameter showed a higher pattern and was significantly higher within the 0.35–0.55 sparsity range for acupuncture manipulation (paired *t*-tests, alpha = 0.05 and statistical power >0.95) (Figure 7B). In addition, other network metrics, including the local efficiency, clustering coefficient, and small-world, did not have a significant difference between acupuncture manipulation and all controls (Supplementary Figure 6).

## Discussion

### A Cooperative PFC-Motor Functional Network for Acupuncture

As the basic acupuncture unit for regulating the central nervous system (Zhang et al., 2012), the Hegu acupoint was employed in this study to explore the neuromodulation effects of acupuncture. Different from most previous findings of studies examining the involvement of subcortical localized brain areas during acupuncture (Chen et al., 2006; Dhond et al., 2008; Yu et al., 2017), our results revealed a distributed and cooperative cortical acupuncture with *deqi* network with the PFC and the motor cortex as nodes.

Firstly, acupuncture manipulation caused significant behavioral *deqi* effects compared to tactile controls (Figure 3). Secondly, by investigating the effects of acupuncture at the Hegu acupoint with *deqi* on cerebral hemodynamic responses, we found the decreased HbO responses could be a potential biomarker for acupuncture (Figure 4 and Supplementary Figure 2). Thirdly, the bilateral PFC and the motor cortex were found to be acupuncture responsive nodes (Figure 5), and the PFC’s hemodynamic responses were significantly correlated with the behavior *deqi* index (Supplementary Figure 3A). Interestingly, the PFC-related connections in the acupuncture network showed significantly increased functional connectivity during acupuncture manipulation (Figure 6 and Supplementary Figure 5). Finally, the topological properties of the acupuncture network showed that the network efficiency was improved by acupuncture with *deqi* (Figure 7). Taken together, these results indicate that there is a cooperative PFC-Motor acupuncture functional network, which could provide more neuroimaging evidence for explaining acupuncture with *deqi*’s neuromodulation effects on the human brain.

### Decreased HbO Response by Acupuncture Manipulation

By analyzing the hemodynamic responses recorded by fNIRS (Figure 4 and Supplementary Figure 1), the bilateral PFC and the motor cortex were found to show decreased HbO responses during acupuncture manipulation (Figure 5 and Supplementary Figure 2). Previous fMRI-fNIRS simultaneous recording studies have proved that the HbO signal is positively correlated with the blood-oxygen-level-dependent (BOLD) fMRI signal (Strangman et al., 2002). In addition, the deactivation of the BOLD signal recorded by fMRI was further proved to reflect the suppression of neuronal activity by previous studies (Shmuel et al., 2002; Stefanovic et al., 2004). These findings suggest that the observed decrease of HbO response from the bilateral PFC and motor cortex could reflect the suppression of underlying neural activities. Moreover, the bilateral PFC and motor cortex could be network nodes during acupuncture manipulation.

### Acupuncture Modulates the PFC and Regulates the Cognition Function

In this study, the bilateral PFC showed significantly decreased HbO responses during acupuncture manipulation (Figure 5 and Supplementary Figure 2), which indicates that acupuncture could modulate the hemodynamic responses of bilateral PFC. The PFC is an important region for high-level cognition processing, which can provide top–down regulation of attention, emotion, and cognitive control (Arnsten and Rubia, 2012). It has been postulated that acupuncture manipulation could regulate cognitive functions by modulating the bilateral PFC responses, as evidenced by previous studies. Firstly, previous studies have shown that the high-level cognitive functions could be modulated by using neuromodulation methods such as TMS (Curtin et al., 2019a), tDCS (Di Rosa et al., 2019), and acupuncture stimulation (Zheng et al., 2018). For example, the previous fNIRS-TMS and fNIRS-tDCS studies found that neuromodulation could improve cognitive performances during working memory tasks (Di Rosa et al., 2019) and speed of processing (SOP) cognitive tasks (Curtin et al., 2019a), which were both evidenced by the reduced amplitude of HbO concentration in the PFC (Curtin et al., 2019a; Di Rosa et al., 2019). Secondly, the dorsolateral prefrontal cortex (DLPFC) is a key node of the central executive network (CEN), which is responsible for high-level cognitive and attentional control (Menon and Uddin, 2010). Therefore, it could be further speculated that the CEN could be modulated by acupuncture manipulation, which helps to improve the brain’s cognitive functions. Finally, previous fMRI studies found that acupuncture modulated BOLD responses and could improve cognition performances for stroke patients (Zhang et al., 2014), mild cognitive impairment patients (Feng et al., 2012), and Alzheimer’s disease (AD) patients (Wang et al., 2014; Zheng et al., 2018), which supported our fNIRS findings. In addition, although acupuncture responsive channels were distributed over the PFC and the motor cortex bilaterally, the PFC showed a left hemisphere dominant response tendency. As all subjects were right-hand controlled in this study, we speculate that it might be related to right-handedness, which needs to be explored in future studies. By modulating bilateral PFC responses, acupuncture manipulation could help regulate cognitive functions.

### The PFC’s HbO Response as an Objective Biomarker for Acupuncture

The needling *deqi* sensation that subjects experienced is considered a valuable behavioral index in evaluating the effect of acupuncture in TCM (Yang et al., 2013). However, the current behavioral evaluation of *deqi* is subjective and insufficient and lacks an objective neural biomarker. In this fNIRS study, we found that the PFC showed a positive correlation trend between the acupuncture *deqi* behavior index and the HbO power change (acupuncture manipulation – all controls) (Supplementary Figure 3), which meant that the PFC’s HbO responses could predict the subject’s behavior performance during acupuncture manipulation. This result showed that the PFC’s HbO response could potentially be used as an objective neural biomarker to evaluate the effects of acupuncture at the Hegu acupoint.

### Modulation of the Motor Cortex and the Analgesic Effect of Acupuncture

In this study, the bilateral motor cortex showed significantly decreased HbO responses during acupuncture manipulation (Figure 5 and Supplementary Figure 2), which indicated that acupuncture could modulate the hemodynamic responses of the bilateral motor cortex. Considering that the motor cortex is an important region for pain processing and control (Volz et al., 2015; Lopes et al., 2019) we argue that by modulating the bilateral motor cortex’s HbO responses, acupuncture manipulation could produce analgesic effects. Firstly, it has been well known that the motor cortex has the role of pain control (Ostergard et al., 2014), and electrical stimulation in the motor cortex could treat intractable pain syndromes effectively (Arle and Shils, 2008; Henssen et al., 2019). Secondly, previous fNIRS studies also proved that analgesic effects could be delivered during acupuncture manipulation, in which the decreased hemodynamic responses of the motor cortex were observed (Takamoto et al., 2010; Rojas et al., 2019). The decreased motor activities showed by the above fNIRS studies were in line with our findings. Taking together, acupuncture manipulation could suppress the excessive motor cortex activity, which helps explain the analgesic effects of acupuncture.

### The Bilateral PFC as Network Hub During Acupuncture

Here, the bilateral PFC was found to have significantly increased functional connections with the motor cortex (PFC-Motor) and with the PFC cortex (PFC-PFC) during acupuncture manipulation compared with all controls (Figure 6 and Supplementary Figure 5). We argue that the bilateral PFC works as a network hub in the cooperative PFC-Motor functional network during acupuncture manipulation. We further postulate that the increased PFC-related functional connectivity could not only improve the human cognitive ability but also produce an analgesic effect through acupuncture manipulation.

Functional connectivity has been widely used to reveal cognition functions (Zeng et al., 2012; Cohen, 2018). Moreover, previous studies showed that the human cognitive ability could be improved during acupuncture with *deqi*, which was evidenced by the increased functional connectivity (Dhond et al., 2008; Bai et al., 2009; Yu et al., 2017). In this study, the observed increased functional connectivity with PFC as node indicated that the long-distance information transmissions with PFC network hub were enhanced. Therefore, acupuncture with *deqi* could strengthen the communications between remote brain areas within the large-scale network and improve cognitive function.

A previous study showed that the PFC-motor’s functional connection had the role of pain control (Peng et al., 2019). In our study, acupuncture manipulation significantly improved the PFC-Motor connections. Therefore, we postulate that the motor cortex might receive increased top-down modulation from the PFC, which could produce the analgesic effect through the recruitment of descending pain inhibition system (Peng et al., 2019). The bilateral PFC is a network hub in the cooperative PFC-Motor functional network for acupuncture. The increased functional connections of bilateral PFC by acupuncture with *deqi* might play a role in improving human cognitive ability and produces analgesic effects.

### Acupuncture Improves the Global Efficiency of the PFC-Motor Functional Network

Compared with all controls, the topological properties of the network showed a lower shortest path length (Figure 7A) and higher global efficiency (Figure 7B) during acupuncture manipulation (Figure 7). We argue that acupuncture manipulation could improve the information transmission of the PFC-Motor functional network. Firstly, global efficiency is a global parameter to describe the network’s capacity for parallel information exchanging between nodes via multiple edges (Rubinov and Sporns, 2010). A higher global efficiency shows that PFC and Motor cortex are well integrated and that information transfer over the PFC-Motor network is more efficient by acupuncture manipulation (Fleischer et al., 2019). Secondly, the path length is a measure of processing steps along the path of information transfer between different nodes (Rubinov and Sporns, 2010). Since fewer numbers of processing steps denote a more rapid and accurate information communication, a lower shortest path length indicates a higher level of communication efficiency among the PFC-Motor network by acupuncture (Kaiser and Hilgetag, 2006).

We further postulate that acupuncture manipulation could improve cognitive ability by improving the global efficiency of the network. Firstly, the functional brain network attained a smaller path length and a larger global efficiency under acupuncture manipulation, indicating that the enhancement of the processing efficiency in the brain network (Yu et al., 2017). Secondly, a previous study found that acupuncture could improve the overall processing efficiency of the brain network among MCI patients, reflecting the improvement of cognitive function by acupuncture manipulation (Ghafoor et al., 2019). Acupuncture manipulation improves the information transmission of the PFC-Motor functional network and could enhance the human cognitive ability, which could also explain the increased functional connections of the PFC-Motor network (Figure 6 and Supplementary Figure 5).

### Limitations and Future Works

There exist several potential limitations to this study. Firstly, more participants should be recruited in future studies to reach a higher statistical power, and provide more evidence for further exploring the relationship between hemodynamic response and acupuncture behavior performance. For example, although the bilateral PFC’s hemodynamic response showed a positive correlation trend with acupuncture behavioral performance (Supplementary Figure 3), the statistical power should be further validated by recruiting more participants in the following study. Secondly, although a quasi-experimental design and pre/post-tactical/rest controls were employed in this study, a more rigorous parallel control experiment design should be conducted in the future, which could make the conclusion more convincing. Thirdly, this study explored the effects of *deqi* on cortical networks, future studies should be conducted with patients to add more neuroimaging evidence for explaining the clinical therapeutic effects of acupuncture with *deqi*. Fourthly, due to the limited channel number of the current fNIRS device, the channel layout of fNIRS could not have complete coverage of the whole brain. In the future, a whole-brain coverage fNIRS device should be used to explore the neural mechanism of acupuncture further. Finally, our study used Pearson’s correlation coefficients as functional connectivity, which could not reveal the causal relationship between brain regions. In future studies, Granger causality analysis could be used to further quantify the strength of directed effective connectivity between brain regions.

## Conclusion

Despite acupuncture’s long history and public acceptance, the underlying neural mechanism of acupuncture’s neuromodulation effects on the human brain remain largely unclear. By using multi-channel fNIRS recordings on the human brain, our study found that acupuncture modulated a distributed and cooperative PFC-Motor cortical network with the bilateral PFC and the motor cortex as key nodes. Taking the basic neural acupuncture unit Hegu acupoint as an example, acupuncture manipulation not only modulated the hemodynamic responses of the bilateral PFC and motor cortex but also regulated the functional connectivity and efficiency of the PFC-Motor cortical network. Our study contributes objective neuroimaging evidence for explaining acupuncture’s neuromodulation effects on the human brain, which also contributes to Traditional Chinese Medicine.

## Data Availability Statement

The data and codes that support the findings of this study are available upon request.

## Ethics Statement

The studies involving human participants were reviewed and approved by the Institutional Review Board and Ethics Committee of Tianjin University. The patients/participants provided their written informed consent to participate in this study.

## Author Contributions

XS designed and conceptualized the research. KZ, LZ, and XS collected the data. SX, SL, LZ, and XS analyzed the data. SX and XS wrote the manuscript. KZ and SL helped with manuscript revision. XS and DM supervision. All authors contributed to the article and approved the submitted version.

## Conflict of Interest

The authors declare that the research was conducted in the absence of any commercial or financial relationships that could be construed as a potential conflict of interest.

## Publisher’s Note

All claims expressed in this article are solely those of the authors and do not necessarily represent those of their affiliated organizations, or those of the publisher, the editors and the reviewers. Any product that may be evaluated in this article, or claim that may be made by its manufacturer, is not guaranteed or endorsed by the publisher.
