# Corrigendum: Acupuncture With *deqi* Modulates the Hemodynamic Response and Functional Connectivity of the Prefrontal-Motor Cortical Network

**DOI:** 10.3389/fnins.2021.773069

**Published:** 2021-11-10

**Authors:** Xiaopeng Si, Shaoxin Xiang, Ludan Zhang, Sicheng Li, Kuo Zhang, Dong Ming

**Affiliations:** ^1^Academy of Medical Engineering and Translational Medicine, Tianjin University, Tianjin, China; ^2^Tianjin Key Laboratory of Brain Science and Neural Engineering, Tianjin University, Tianjin, China; ^3^Tianjin International Engineering Institute, Tianjin University, Tianjin, China; ^4^Institute of Applied Psychology, Tianjin University, Tianjin, China

**Keywords:** acupuncture, functional connectivity, functional near-infrared spectroscopy, motor cortex, prefrontal cortex

In the original article, there was a mistake in the Supplementary Figure S2 as published. In the title of Supplementary Figure S2A “Rest control” should be changed to “All controls”. The correct version of Supplementary Figure S2 appears below.

**Supplementary Figure S2:**
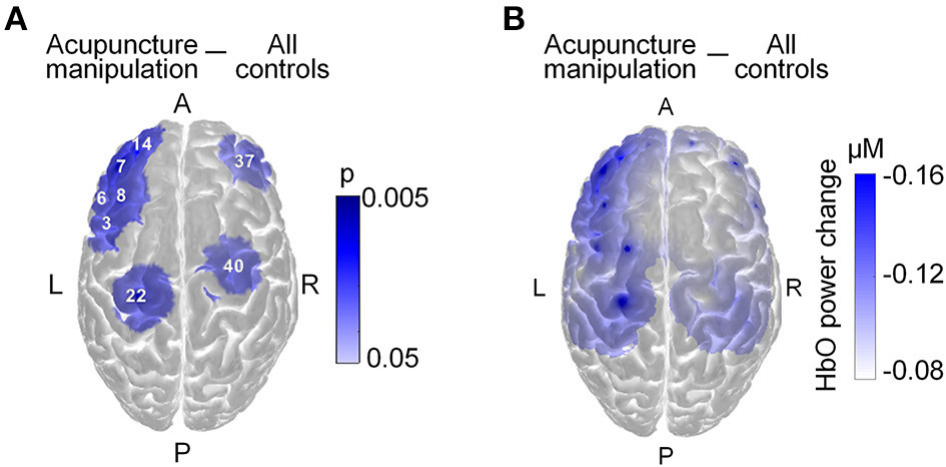
Acupuncture responsive areas. **(A)** Locations and mapping of the significantly decreased acupuncture responsive channels (*n* = 8, the digital number for acupuncture response channel, two-sample *t*-tests, FDR correction for multiple comparisons, alpha = 0.05 and statistical power > 0.95, visualized by *p*-value, blue color for decrease). **(B)** Mapping of the group-averaged HbO power change for acupuncture manipulation vs. all controls.

The authors apologize for this error and state that this does not change the scientific conclusions of the article in any way. The original article has been updated.

## Publisher's Note

All claims expressed in this article are solely those of the authors and do not necessarily represent those of their affiliated organizations, or those of the publisher, the editors and the reviewers. Any product that may be evaluated in this article, or claim that may be made by its manufacturer, is not guaranteed or endorsed by the publisher.

