# Supplementary material for: Acupuncture With deqi Modulates the Hemodynamic Response and Functional Connectivity of the Prefrontal-Motor Cortical Network
Source: Front Neurosci. 2021 Aug 16;15:693623. doi: 10.3389/fnins.2021.693623 (PMC8415569; doi:10.3389/fnins.2021.693623)
Supplement: Supplementary file 1 [file Data_Sheet_1.pdf]

Supplementary Material

Title

Acupuncture with *deqi* Modulates the Hemodynamic Response and Functional Connectivity of the Prefrontal-Motor Cortical Network

Authors

Xiaopeng Si,[1,2,3,4] Shaoxin Xiang,[2,3] Ludan Zhang,[1,2] Sicheng Li,[1,2] Kuo Zhang,[1,2,*] and Dong Ming[1,2,*]

Supplementary information	The details in Materials and Methods
Supplementary Figure S1	fNIRS pre-processing procedure
Supplementary Figure S2	Acupuncture responsive areas
Supplementary Figure S3	Correlation analysis between hemodynamic response (HbO power change) and acupuncture's behavior performance (*deqi* index)
Supplementary Figure S4	Matrices of significant functional connectivity for different conditions
Supplementary Figure S5	Statistical comparisons of the functional connectivity changes of different region pairs between acupuncture manipulation and all controls
Supplementary Figure S6	Statistical comparations of global network metrics for acupuncture manipulation vs. all controls with different sparsity thresholds
Supplementary Table S1	Each subject's *deqi* sensation behavior scores for acupuncture manipulation and pre/post-manipulation tactile controls

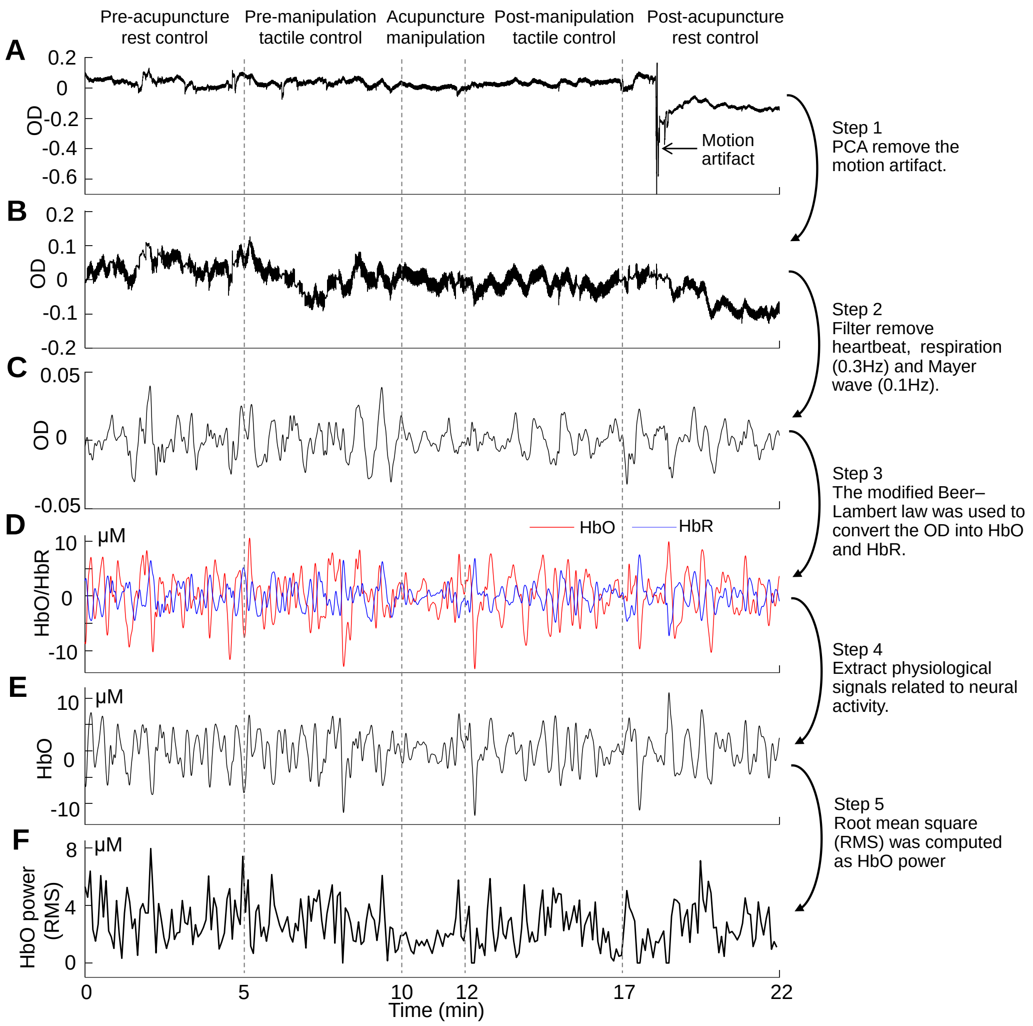

Supplementary Figure S1. fNIRS pre-processing procedure. (A) The optical density (OD) raw data. Arrow for motion artifacts. (B) Motion artifact corrected OD data by principal component analysis (PCA). (C) The filtered OD data to remove physiological noise. (D) Original HbO and HbR concentration data. (E) The neural activity related HbO data. (F) The root mean square (RMS) power of HbO. Procedure details: Step 1, PCA was used to remove motion artifacts. Step 2, 0.01-0.2 Hz bandpass filter to remove heartbeat (~1 Hz) and respiration (~0.3 Hz); 0.1 Hz notch filter to remove Mayer wave (~0.1 Hz). Step 3, the modified Beer–Lambert law was applied to convert OD into HbO and HbR. Step 4: Physiological signals related to neural activity were extracted from original HbO and HbR. Step 5: The RMS of HbO was computed as HbO power.

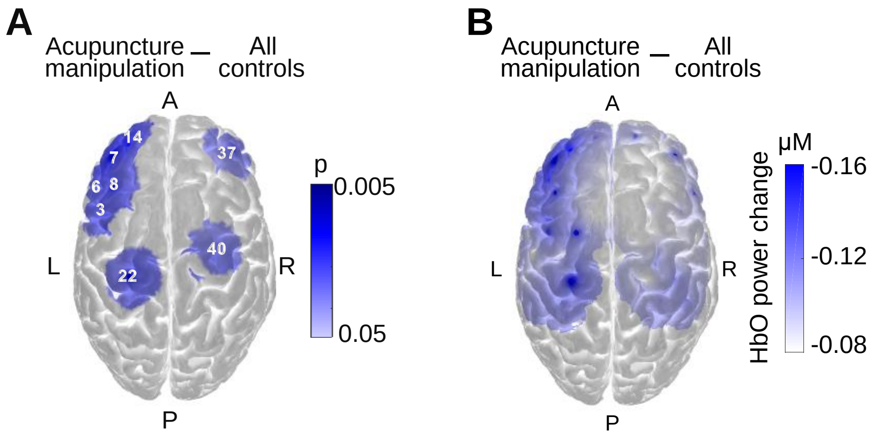

Supplementary Figure S2. Acupuncture responsive areas. (A) Locations and mapping of the significantly decreased acupuncture responsive channels (n = 8, the digital number for acupuncture response channel, two-sample t-tests, FDR correction for multiple comparisons, alpha = 0.05 & statistical power > 0.95, visualized by p-value, blue color for decrease). (B) Mapping of the group-averaged HbO power change for acupuncture manipulation vs. all controls.

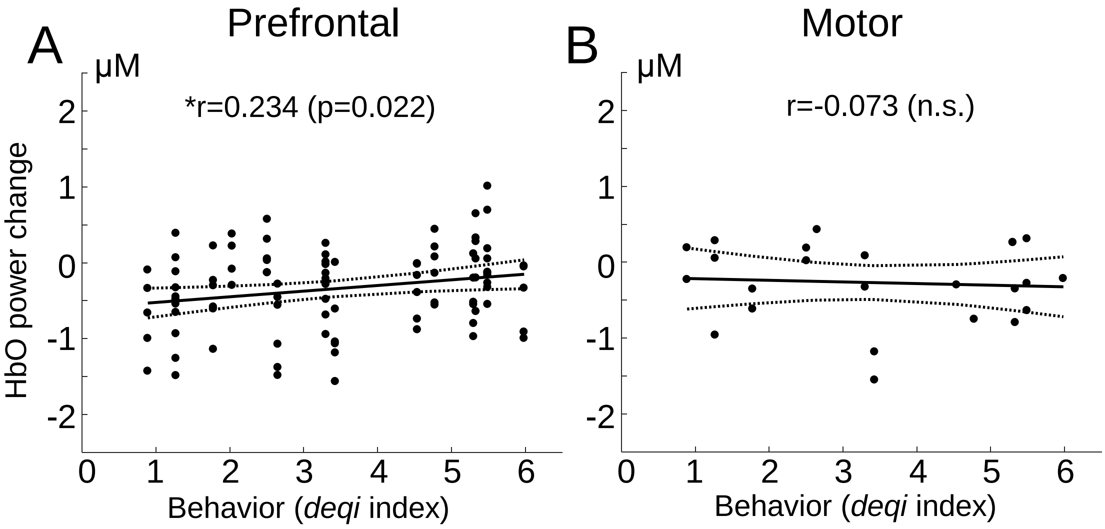

Supplementary Figure S3. Correlation analysis between hemodynamic response (HbO power change) and acupuncture's behavior performance (*deqi* index). For acupuncture responsive areas: (A) the PFC and (B) the motor cortex, respectively. (Pearson's correlations, * p < 0.05 & statistical power > 0.50, n.s. for non-significance, dashed line for 95% confidence intervals of the polynomial evaluated at each *deqi* index). Dots represented for the significant acupuncture responsive channels within the prefrontal (A) or motor (B) cortex from each individual subject. (The label μM means the unit of HbO power change).

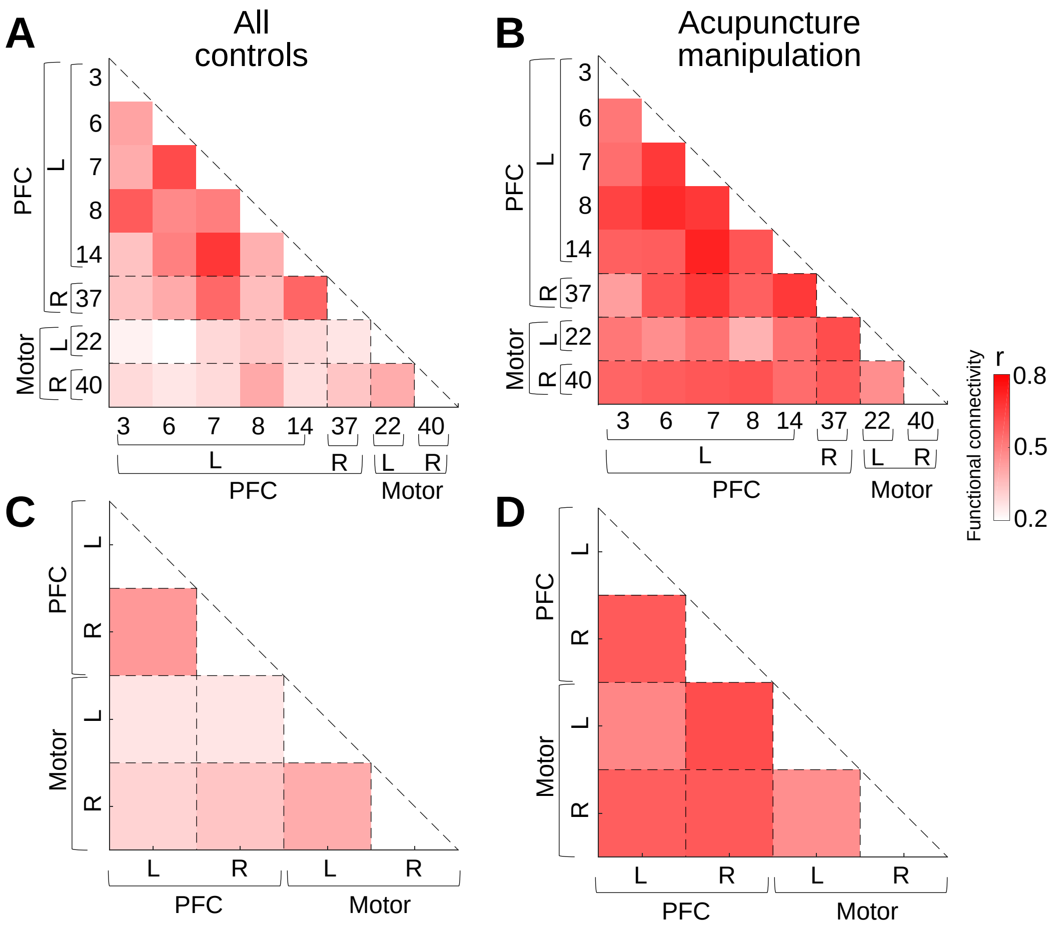

Supplementary Figure S4. Matrices of significant functional connectivity for different conditions. (A and B) Group level's channel-to-channel functional connectivity matrices of all acupuncture responsive channel pairs for (A) all controls and (B) acupuncture manipulation (permutation test, $p < 0.001$). (C and D) Group level's region-to-region functional connectivity matrices between different acupuncture responsive cortical regions for (C) all controls and (D) acupuncture manipulation. PFC = prefrontal cortex, R=right, L=left.

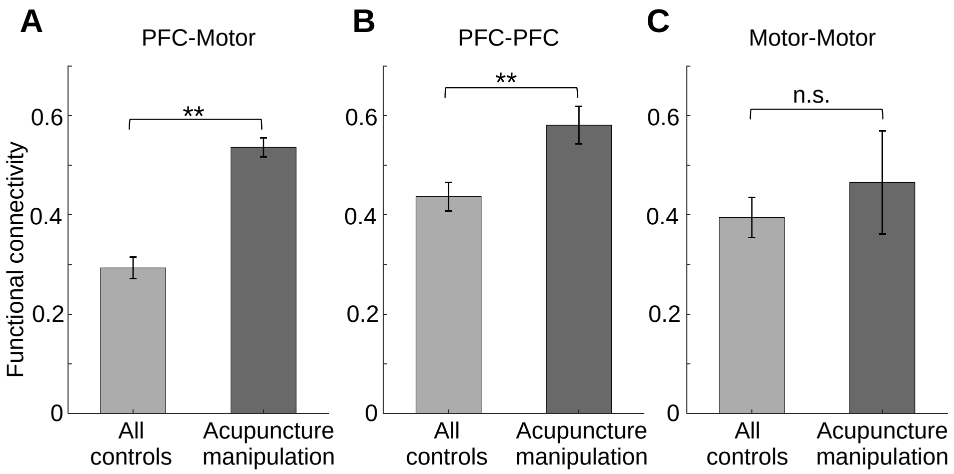

Supplementary Figure S5. Statistical comparisons of the functional connectivity changes of different region pairs between acupuncture manipulation and all controls. Functional connectivity changes comparisons for (A) PFC-Motor, (B) PFC-PFC, and (C) Motor-Motor (mean±SEM, paired t-tests, ** $p < 0.01$ & statistical power > 0.95, n.s. for non-significance; PFC = prefrontal cortex).

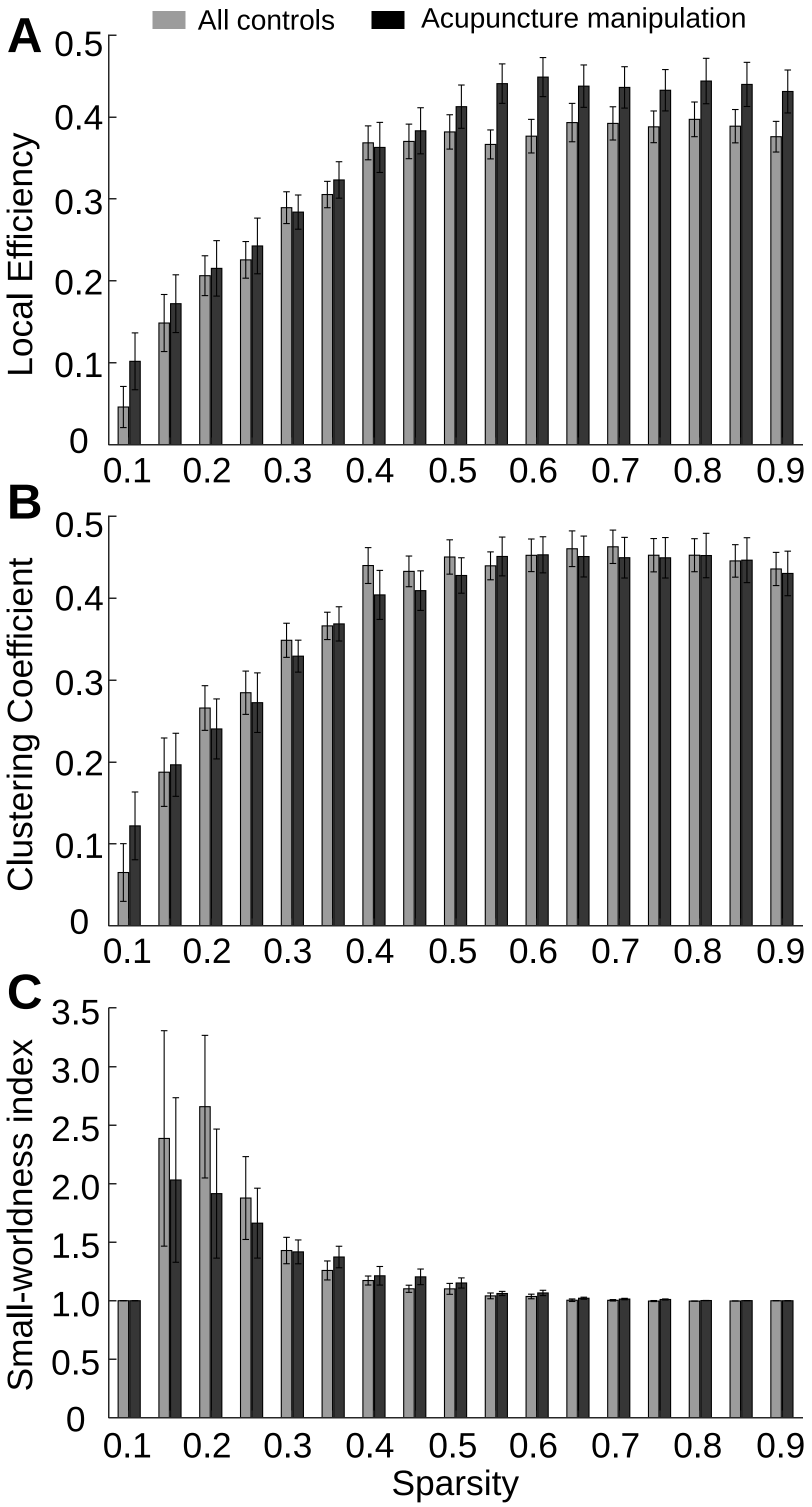

Supplementary Figure S6. Statistical comparations of global network metrics for acupuncture manipulation vs. all controls with different sparsity thresholds. (A) Local Efficiency. (B) Clustering Coefficient. (C) Small-worldness index. Note: paired t-tests, non-significance, subjects, n = 17, sparsity range = [0.1, 0.9], sparsity step-size = 0.05.

Supplementary Table S1. Each subject's *deqi* sensation behavior scores for acupuncture manipulation and pre/post-manipulation tactile controls.

Subject	Condition	Soreness	Numbness	Distention	Heaviness	Spread	Pain	*deqi* index
	Control (pre)	1	1	2	2	3	1	2.4
S1	Acupuncture	1	3	3	1	1	2	2.65
	Control (post)	1	2	1	2	3	1	2.4
	Control (pre)	4	2	4	2	0	2	3.49
S2	Acupuncture	5	4	2	2	2	5	4.54
	Control (post)	4	2	4	1	2	4	3.76
	Control (pre)	2	0	0	2	2	1	1.84
S3	Acupuncture	0	7	3	5	3	3	5.49
	Control (post)	5	2	5	4	2	3	4.6
	Control (pre)	3	0	0	0	0	0	1.52
S4	Acupuncture	1	0	0	0	0	2	1.27
	Control (post)	0	0	0	0	0	0	0
	Control (pre)	3	3	4	3	4	4	3.89
S5	Acupuncture	5	4	5	5	5	6	5.49
	Control (post)	3	3	4	3	4	3	3.76
	Control (pre)	2	4	4	2	5	4	4.41
S6	Acupuncture	5	3	5	3	5	3	4.78
	Control (post)	3	4	5	3	4	3	4.4
	Control (pre)	2	0	5	0	6	3	4.83
S7	Acupuncture	2	4	4	0	7	3	5.33
	Control (post)	0	0	0	0	2	0	1.02
	Control (pre)	0	0	0	0	0	0	0
S8	Acupuncture	3	0	4	2	0	3	3.3
	Control (post)	3	2	3	1	0	1	2.63
	Control (pre)	1	1	2	1	1	1	1.51
S9	Acupuncture	3	2	2	2	2	2	2.51
	Control (post)	1	1	2	1	1	1	1.51
	Control (pre)	2	0	2	0	2	0	1.78
S10	Acupuncture	3	2	5	3	8	1	5.98
	Control (post)	2	4	3	3	5	1	4.21
	Control (pre)	3	3	5	2	2	2	3.9
S11	Acupuncture	5	5	6	2	2	4	5.3
	Control (post)	3	3	4	2	1	2	3.38
	Control (pre)	0	2	5	0	0	0	3.05
S12	Acupuncture	0	5	3	0	0	1	3.43
	Control (post)	0	2	2	0	0	0	1.52
	Control (pre)	0	0	2	0	0	0	1.02
S13	Acupuncture	4	0	0	0	0	0	2.03
	Control (post)	2	0	0	0	0	0	1.02
S14	Control (pre)	2	3	2	1	5	2	3.76
	Acupuncture	3	3	1	1	2	1	2.65

	Control (post)	2	4	3	1	5	1	4.11
	Control (pre)	0	0	0	0	0	2	1.02
S15	Acupuncture	0	2	2	0	0	2	1.78
	Control (post)	0	1	1	0	0	2	1.4
	Control (pre)	0	0	3	0	0	0	1.52
S16	Acupuncture	0	0	4	0	4	2	3.3
	Control (post)	0	0	2	0	0	0	1.02
	Control (pre)	0	0	0	0	0	0	0
S17	Acupuncture	2	0	0	0	0	1	1.27
	Control (post)	0	0	0	0	0	0	0
	Control (pre)	0	1	0	1	1	2	1.46
S18	Acupuncture	0	2	0	1	0	0	1.27
	Control (post)	0	1	0	1	0	0	0.76
	Control (pre)	1	0	2	2	0	1	1.71
S19	Acupuncture	1	0	1	1	0	0	0.89
	Control (post)	0	0	1	0	0	0	0.51
	Control (pre)	2	4	1	0	7	1	4.92
S20	Acupuncture	1	5	1	0	4	1	3.78
	Control (post)	2	5	1	0	6	1	4.67

Note: Control (pre) = pre-manipulation tactile control, Acupuncture = acupuncture manipulation, Control (post) = post-manipulation tactile control.
